# Delayed repair for aortic root abscess - is it justified?

**DOI:** 10.1186/1749-8090-10-S1-A234

**Published:** 2015-12-16

**Authors:** Vasudev B Pai, Ganesh S Kamath, Guruprasad D Rai, Muralidhar Varma

**Affiliations:** 1 Department of CVTS, Kasturba Medical College, Manipal University, Manipal, Karnataka, 576104, India; 2 Department of Medicine, Kasturba Medical College, Manipal University, Manipal, Karnataka, 576104, India

## Background/Introduction

Surgery is indicated in patients with infective endocarditis with a root abscess.

## Aims/Objectives

To describe a case report of delayed surgery for aortic root abscess.

## Method

We present a patient of endocarditis with a root abscess who was operated after a few weeks due to other medical reasons.

## Results

A 55 yr old gentleman presented with high grade fever since 2mths and dyspnoea on exertion since 1mth. Echocardiography revealed severe AR with vegetations and a diagnosis of infective endocarditis was made after blood cultures grew coagulase negative staphylococcus. He was in pulmonary oedema on presentation with renal failure needing non-invasive ventilator support and low dose inotropes. He responded to appropriate intravenous antibiotics. He was investigated for malaena and CT scan showed splenomegaly with cirrhosis suggestive of portal hypertension and there was suspicion of an aortic root abscess. Upper GI scopy showed oesophageal varices. He was referred for aortic valve replacement. Repeat transoesophageal echocardiograms did not show the root abscess and his renal dysfunction contraindicated repeat CT scans. He was taken up for banding for varices and surgery was delayed for a month. The inflammatory markers returned to normal; renal function recovered to normal and heart failure resolved as well. Appropriate antibiotic cover was continued for the 4 weeks after banding. An elective aortic valve replacement was planned with a tissue valve considering the varices. At surgery a cavity of 0.5 cm × 0.5 cm × 1 cm was found between the LCC- RCC junction medial to the pulmonary artery. The annulus was reconstructed with obliteration of the cavity. He made a good recovery thereafter.

## Discussion/Conclusion

Guidelines indicate that surgery is indicated in patients with endocarditis having root abscesses. This patient had a root abscess which was controlled with antibiotics and surgery was delayed for other medical reasons. We conclude that guideline based decision making is useful but there are times when a slightly delayed intervention might show good results especially in patients who respond to medical treatment even with good indications to operate.

## Consent

Written informed consent was obtained from the patient's next of kin for publication of this abstract and any accompanying images. A copy of the written consent is available for review by the Editor of this journal.

